# A global virus network and a perspective on viral infections in humans

**DOI:** 10.1186/1742-4690-9-S1-I22

**Published:** 2012-05-25

**Authors:** Robert Gallo

**Affiliations:** 1 Institute of Human Virology, Baltimore, USA

## 

During the flu pandemic of the 1910s, the polio outbreaks of the 1950s, and the AIDS crisis of the 1980s, invaluable time was wasted and a large number of people died or became seriously ill while authorities planned a response, identified and concentrated resources, and developed a plan to prevent others from becoming infected.

Each of these 3 pandemics of the last century as well as the smallpox epidemics were caused by viruses. I have noted that often there are no expert responsible medical virologists, and concomitantly I noted a decline in newly trained medical virologists.

Without experts, initial responses to viral outbreaks can be needlessly harsh and economically devastating. Fear replaces logical analysis, and entire populations of healthy pigs and birds have been slaughtered simply because they could potentially harbor swine or avian flu strains. These over-reactions hurt small farmers and the economies in developing nations, and make crafting a vaccine more difficult.

Public health authorities require a reliable source of experts on basic human and animal virology to turn to for rapid answers. A select group of frontline virologists is needed to work quickly to convert preliminary information into advances in the laboratory to protect the blood supply when needed and begin looking for clinical treatments and, ultimately, a vaccine.

Organizations involved in the surveillance of viruses, like the World Health Organization and CDC, and those involved in the delivery of care during emergencies, like the Gates Foundation’s GAVI Program, often do not have the manpower for training initiatives and basic virus research which are important parts of addressing any public health threat. The Global Virus Network is uniquely positioned to utilize the expertise of its members to bridge these efforts.

The overall aim of the network is to achieve accelerated, innovative solutions to human viral diseases. The GVN also focuses on coordinated virology training for junior scientists and developing scholar exchange programs for recruiting and training the next generation of medical virologists.

Today, the GVN is made up of 30 centers and 3 affiliates in 19 countries on 6 of the world’s 7 continents. Its membership includes all 7 medical virologists in the U.S. National Academy of Sciences, and others who are members of their own countries’ national academies. Their collective expertise covers all types of existing, emerging, and re-emerging viruses which affect humans.

